# Surgical Management and Outcomes following Pathologic Hip Fracture—Results from a Propensity Matching Analysis of the Registry for Geriatric Trauma of the German Trauma Society

**DOI:** 10.3390/medicina58070871

**Published:** 2022-06-29

**Authors:** Christopher Bliemel, Katherine Rascher, Ludwig Oberkircher, Torsten Schlosshauer, Carsten Schoeneberg, Matthias Knobe, Bastian Pass, Steffen Ruchholtz, Antonio Klasan, on behalf of the AltersTraumaRegister DGU

**Affiliations:** 1 Center for Orthopaedics and Trauma Surgery, University Hospital Giessen-Marburg, Baldingerstrasse, 35043 Marburg, Germany; ruchholt@med.uni-marburg.de; 2 Akademie der Unfallchirurgie GmbH, 80538 Munich, Germany; katherine.rascher@auc-online.de; 3 Department of Trauma Surgery, Orthopaedics and Arthroplasty, Hospital Friedrichshafen, 88048 Friedrichshafen, Germany; l.oberkircher@klinikum-fn.de; 4 Department of Plastic Surgery, Agaplesion Evangelical Hospital Giessen, 35398 Gießen, Germany; torsten.schlosshauer@ekm-gi.de; 5 Department of Orthopedic and Emergency Surgery, Alfried Krupp Hospital Essen, 45131 Essen, Germany; carsten.schoeneberg@krupp-krankenhaus.de (C.S.); bastian.pass@krupp-krankenhaus.de (B.P.); 6 Department of Orthopaedic and Trauma Surgery, Cantone Hospital Lucerne,6004 Luzern, Switzerland; matthias.knobe@luks.ch; 7 Department for Orthopaedics and Traumatology, Kepler University Hospital Linz, 4020 Linz, Austria; klasan.antonio@me.com

**Keywords:** pathologic femoral fracture, outcome, mortality, mobility, AltersTraumaRegister DGU^®^

## Abstract

*Background and Objectives:* The outcomes of patients with pathologic hip fractures remain unclear. Data from a large international geriatric trauma registry were analyzed to examine the outcomes of patients with pathologic hip fractures compared with patients with typical osteoporotic hip fractures. *Materials and Methods:* Data from the Registry for Geriatric Trauma of the German Trauma Society (Deutsche Gesellschaft für Unfallchirurgie (DGU)) (ATR-DGU) were analyzed. All patients treated surgically for osteoporotic or pathologic hip fractures were included in this analysis. Across both fracture types, a 2:1 optimal propensity score matching and multivariate logistic regression analysis were conducted. In-house mortality rate and mortality at the 120-day follow-up, as well as mobility after 7 and 120 days, reoperation rate, discharge management from the hospital and readmission rate to the hospital until the 120-day follow-up were analyzed as outcome parameters for the underlying fracture type—pathologic or osteoporotic. *Results:* A total of 29,541 cases met the inclusion criteria. Of the patients included, 29,330 suffered from osteoporotic fractures, and 211 suffered from pathologic fractures. Multivariate logistic regression analysis revealed no differences between the two fracture types in terms of mortality during the acute hospital stay, reoperation during the initial acute hospital stay, walking ability after seven days and the likelihood of being discharged back home. Walking ability and hospital readmission remained insignificant at the 120-day follow-up as well. However, the odds of passing away within the first 120 days were significantly higher for patients suffering from pathologic hip fractures (OR: 3.07; *p* = 0.003). *Conclusions:* Surgical treatment of pathologic hip fractures was marked by a more frequent use of arthroplasty in per- and subtrochanteric fractures. Furthermore, the mortality rate among patients suffering from pathologic hip fractures was elevated in the midterm. The complication rate, as indicated by the rate of readmission to the hospital and the necessity for reoperation, remained unaffected.

## 1. Introduction

In comparison to traumatic bone fractures, pathologic fractures due to diseased bone are less common events. A pathologic fracture is one that occurs without adequate trauma and is in most cases caused by a malignant bony lesion. Apart from primary malignant osseous tumors, osseous metastasizing carcinomas of the lung, breast, kidney, thyroid gland and prostate are responsible for the vast majority of bony lesions [1]. Apart from prostate metastases, which are usually osteoblastic, bony lesions mainly appear as lytic or mixed.

Due to a very well-developed vascular system in the intertrochanteric region, bony metastases are particularly common in the area of the proximal femur [2,3]. This circumstance favors pathologic fractures of the hip, as the mechanical loading stress during walking, which is transferred from the pelvic ring on to the femoral shaft, is extremely high [4,5,6,7,8].

As the vast majority of geriatric hip fractures are known to be related to osteoporosis rather than cancer, it is scarcely surprising that most of the literature focuses on this primary cause [9,10,11]. Such findings on geriatric hip fractures have already been included in national guidelines for several years and are further implemented as quality indicators in the treatment of geriatric hip fracture patients [12,13].

Despite the overlap between both patient groups with regard to fracture site and therapeutic goals, such as pain relief, mobilization or maintenance of patients’ independence, it remains unclear whether the findings derived from osteoporotic hip fractures can be transferred one-to-one to patients with pathologic hip fractures.

Currently, the literature on this topic remains limited and contradictory. Some studies report similarities between both groups of patients, especially in terms of the occurrence of perioperative complications, such as pneumonia, wound healing disorders and sepsis [2] or in the rate of total hip arthroplasties (THAs) performed [14]. On the other hand, discordant findings were found in other studies, such as the sex rate of patients affected [15], the comorbidity profile of patients [14] or the outcome related to delay in time to surgery [16].

To provide more clarity regarding outcomes of patients with pathologic and non-pathologic fractures, we made use of the data contained in the Registry for Geriatric Trauma (AltersTraumaRegister DGU^®^ (ATR-DGU)) of the German Trauma Society (Deutsche Gesellschaft für Unfallchirurgie (DGU)).

It was hypothesized that, compared to osteoporosis-related hip fractures, the presence of metastasis-related hip fractures would lead to increased rates of perioperative complications and mortality among those patients with pathologic fractures.

## 2. Materials and Methods

This study is a retrospective cohort registry study comparing patients with malignant, pathologic fractures vs. patients with non-pathologic (osteoporotic) fractures. All patient data were obtained from the ATR-DGU.

### 2.1. ATR-DGU

The source of the data in the present analysis is the ATR-DGU (http://www.alterstraumaregister-dgu.de (accessed on 29 November 2021). The ATR-DGU was established in 2016 by the German Trauma Society (DGU). It is a large, prospective, multicenter, standardized registry that provides information on geriatric trauma patients with hip, periprosthetic and peri-implant femoral fractures. The reliability of ATR-DGU has already been shown elsewhere [17]. All DGU-certified AltersTraumaZentren (Specialty Orthogeriatric Departments) are required to enter patient data into the ATR-DGU. Data entry was only possible with consent of the patient. Therefore, all patients who did not sign a consent form were excluded. Participating centers transmit pseudonymized patient data via a web-based application into a central database. Currently, approximately 120 hospitals from Germany, Switzerland and Austria contribute to the ATR-DGU. The scientific management of the ATR-DGU is carried out by the Working Committee on Geriatric Trauma Registry (AK ATR) of the DGU. Approval for scientific data analysis from the ATR-DGU is granted via a peer-review process in accordance with the publication guidelines laid out by the AK ATR. The present study is in accordance with the publication guidelines of the ATR-DGU and registered as ATR-DGU project ID 2021-007. The inclusion criteria of the ATR-DGU are patients with proximal femur fractures, including periprosthetic and peri-implant fractures requiring surgery, who are aged 70 years or older. The ATR-DGU collects data in five distinct phases: pre-injury, intake, surgery, first week post-surgery and an optional 120-day follow-up [18].

### 2.2. Inclusion and Exclusion Criteria

This study analyzed 34,895 patients documented in the registry from 2016 to 2020. Patients with periprosthetic and peri-implant fractures were excluded, as well as atypical femoral fractures and fractures of unknown entity. This resulted in an initial analysis group of 29,541 patients from 119 hospitals. Two patient groups were compared—those with malignant, pathologic fractures vs. patients with non-pathologic (osteoporotic) fractures. Outcome parameters were mortality during the acute hospital stay and until the 120-day follow-up, reoperation rate during the initial hospital stay, walking ability 7 and 120 days after surgery, living situation after release from the hospital and readmission to the hospital during the follow-up phase.

### 2.3. Statistical Analysis

To control for differences between the demographics of the two groups, a 2:1 optimal propensity score matching was conducted. Matching was performed using the MatchIt package [19] in R v. 4.0.2 (Foundation for Statistical Computing, Vienna, Austria), which uses functions from the optmatch package [20]. The covariates used in the matching were age, sex, American Society of Anesthesiologists (ASA) score, type of fracture and walking ability before fracture. After matching, the absolute standardized mean differences of all covariates were less than 0.08, indicating that good balance was achieved.

For descriptive analyses, categorical data are presented as counts and percentages, and continuous variables are presented as the means with standard deviation (sd). Comparisons between patient groups were made using the χ²-test for categorical variables and the Mann–Whitney test for continuous variables. Furthermore, logistic and linear regressions were performed on the matched dataset to test for differences in the above-listed outcome parameters. All differences were considered statistically significant when *p* < 0.05.

### 2.4. Aim of the Study and Outcome Parameters

**Figure 1:**
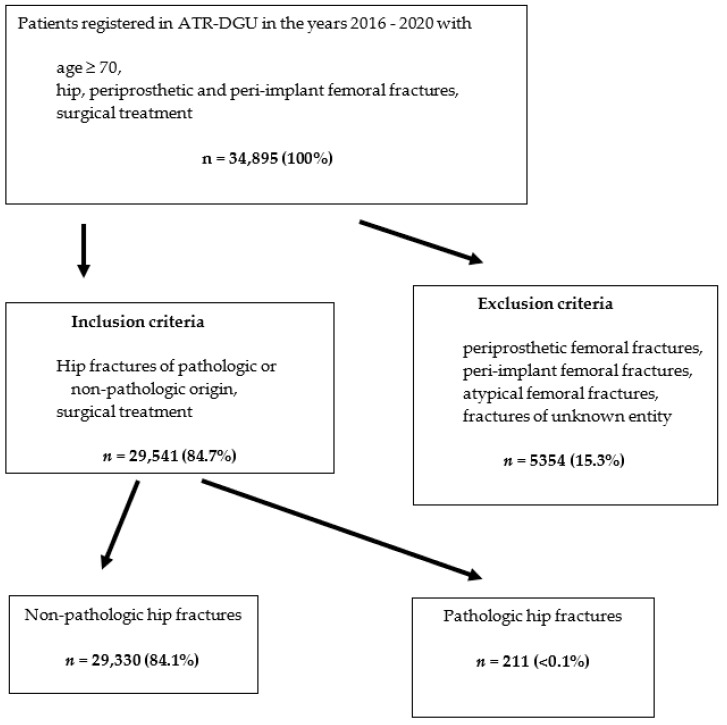
Flow sheet of the included population.

The aim of the study was to analyze the differences in complication and mortality rates during the acute hospital stay and at the 120-day follow-up, depending on the fracture type—pathologic or non-pathologic (osteoporotic). Univariable outcomes were examined separately for patients who suffered from non-pathologic and pathologic hip fractures (Figure 1). Other outcomes studied were the mobility of patients, their reoperation rate and discharge management, as well as the rate of readmission to the hospital within the first 120 days following the initial surgical treatment.

The present analysis covered the following data: sex, age, ASA score, Identification of Seniors At Risk (ISAR) score [21], residential status (before the fracture and at 120-day follow-up), fracture type, anticoagulation on admission, time to surgery, type of surgical treatment, surgical complication (120-day follow-up), walking ability (on day 7 after surgery and at 120-day follow-up), discharge after hospital and mortality (at the initial stay and at 120-day follow-up).

## 3. Results

### 3.1. Acute Care Data

A total of 29,541 hip fractures from geriatric trauma patients met the inclusion criteria. Of these fractures, 29,330 fractures were of non-pathologic origin, and 211 fractures were of pathologic origin.

Univariable data analysis in terms of the fracture origin (non-pathologic or pathologic) is shown in Table 1. This analysis revealed that patients with pathologic femoral fractures had a more balanced sex distribution (*p* < 0.001) and were younger in age (*p* < 0.001) than those with non-pathologic femoral fractures. Further differences were seen in the ASA score and time to surgery, with patients suffering from pathologic fractures having increased ASA scores (*p* < 0.001) and a delay in surgical stabilization (*p* = 0.002). Representing approximately a quarter of cases, subtrochanteric fractures were much more common in patients with pathologic fractures (*p* < 0.001). Patients with pathologic femoral fractures were also more likely to have an independent residential status before the fracture (*p* < 0.001) and were discharged home more often (*p* = 0.002).

Due to such differences in the demographics of the baseline parameters in both patient groups, an optimal propensity score matching analysis was performed, as illustrated in Table 2. Based on a 2:1 matching of 382 patients with non-pathologic fractures and 191 patients with pathologic fractures, there was a significant delay in time to surgery for patients with pathologic fractures (*p* = 0.005). Additionally, there were significant differences in the type of surgical treatment for per- and subtrochanteric fractures, with pathologic fractures being more often treated by arthroplasty compared to non-pathologic femoral hip fractures (*p* = 0.002).

After controlling for age, sex, ASA score, type of fracture and walking ability before fracture, no differences were found between patients with pathologic and non-pathologic hip fractures regarding death during the acute hospital stay (*p* = 0.155), the reoperation rate during the acute hospital stay (*p* = 0.314), the walking ability after seven days (*p* = 0.856) or being discharged back home rather than to an inpatient facility (*p* = 0.295) (Table 3).

### 3.2. 120-Day Follow-Up Data

For 12,887 patients with non-pathologic hip fractures and 86 patients with pathologic hip fractures, data are available at the time of the 120-day follow-up (Table 4).

Patients suffering from pathologic fractures had a significantly higher mortality rate within the first 120 days following surgery compared to non-pathologic hip fracture patients (31% vs. 11%; *p* = 0.001). Other parameters, such as walking ability (*p* = 0.588), place of residence (*p* = 0.965), preoperative vs. postoperative change in residential status (*p* = 0.988) and the rate of readmission or reoperation during the follow-up period (*p* = 0.648 and *p* = 0.374), were comparable between both fracture types (Table 4).

Based on a 2:1 matching, 138 non-pathologic hip fracture patients were compared to 84 patients with pathologic hip fractures. Trends in the matched data were the same as those in the unmatched data. Mortality was significantly higher in the pathologic fracture group than in the non-pathologic fracture group (*p* < 0.001). In contrast, place of residence did not differ significantly across the two fracture groups (*p* = 0.965). Similarly, there were no significant differences in patients’ ability to walk (*p* = 0.627), the preoperative vs. postoperative change in residence (*p* = 0.903) or the rate of readmission or reoperation during the follow-up period (*p* = 0.920 and *p* = 0.725; Table 5).

Multivariate analysis of parameters collected at follow-up showed that the odds ratio for dying within 120 days postoperatively was significantly higher in patients with pathologic fractures (OR: 3.07; *p* = 0.003; Table 3). However, the 120-day readmission rate and patients’ walking ability did not differ between patients with non-pathologic and pathologic fractures (*p* = 0.683 and *p* = 0.396) (Table 3).

**Table 3:** Multivariable logistic regression analysis—pathologic vs. non-pathologic femur fracture. Analysis is adjusted for sex, patient age, ASA score, fracture type and pre-fracture walking ability. The model “discharge from hospital” is adjusted to the pre-fracture living situation.

Influence of the Fracture Entity on…	N	OR	95%-CI and OR	*p*-Value
Acute phase				
Death during stay in the acute hospital * Yes vs. no	573	1.57	[0.83; 2.92]	0.155
Reoperation during initial acute hospital stay * Yes vs. no	573	1.63	[0.61; 4.19]	0.314
Walking ability after seven days * able to walk vs. not able/only at home	557	0.96	[0.62; 1.51]	0.856
Discharge from hospital back home * Yes vs. no	519	1.25	[0.82; 1.91]	0.295
120-day follow-up				
Mortality during follow-up * Yes vs. no	222	3.07	[1.46; 6.47]	0.003
Readmission to hospital during follow-up * Yes vs. no	213	1.28	[0.39; 4.18]	0.683
Walking ability after 120 days * able to walk vs. not able/only at home	175	0.64	[0.23; 1.86]	0.396

* Logistic regression.

**Table 4:** Univariable analysis of 120-day follow-up data on geriatric trauma patients with hip fractures depending on the kind of fracture entity.

Parameter		Non-Pathologic Fracture	Pathologic Fracture	*p*-Value
Number of patients		12,887	86	
Ability to walk	Without aid	1044 (10.9%)	4 (7.0%)	0.588 *
	With walking stick or crutch	1153 (12.1%)	9 (15.8%)	
	With two crutches or a rollator	4069 (42.6%)	28 (49.1%)	
	Certain ability to walk indoors	2020 (21.1%)	9 (15.8%)	
	Not possible	1270 (13.3%)	7 (12.3%)	
Residential status	At home\assisted living facility	6008 (67.1%)	36 (76.6%)	<0.361 *
	Nursing home	2768 (30.9%)	10 (21.3%)	
	Hospital\Inpatient Facility	178 (2.0%)	1 (2.1%)	
120-day mortality	Dead	1122 (11%)	21 (30.9%)	<0.001 *
Changes in living situation at 120-day follow-up	Pre-fracture living at home and still living at home	5666 (82.4%)	34 (82.9%)	0.988 *
	Pre-fracture living at home changed to nursing home	1056 (15.4%)	6 (14.6%)	
	Pre-fracture living at home changed to other inpatient facility	152 (2.2%)	1 (2.4)	
Readmission to hospital during follow-up	Yes	569 (4.6%)	5 (6.3%)	0.648 *
	No	11,774 (95.4%)	74 (93.7%)	
Reoperation during follow-up	Yes	469 (4.0%)	5 (6.7%)	0.374 *
	No	11,315 (96.0%)	70 (93.3%)	
Type of reoperation +	Conversion into total hip arthroplasty	81	0	
	Conversion into hemiarthroplasty	51	0	
	Girdlestone situation	9	0	
	Periprosthetic fracture/peri-implant fracture	42	0	
	Implant removal	84	0	
	Reposition	45	2	
	Revision of osteosynthesis	57	1	
	Irrigation or debridement	130	2	
	Other	115	1	

* Chi-Square Test; + multiple choices possible.

**Table 5:** Univariable analysis of a 2:1 optimal propensity score matching analysis of 120-day follow-up data on geriatric trauma patients with hip fractures depending on the kind of fracture entity.

Parameter		Non-Pathologic Fracture	Pathologic Fracture	*p*-Value
Number of patients		138	84	
Ability to walk	Without aid	13 (10.9%)	4 (7.1%)	0.627 *
	With walking stick or crutch	14 (11.8%)	9 (16.1%)	
	With two crutches or a rollator	55 (46.2%)	27 (48.2%)	
	Certain ability to walk indoors	27 (22.7%)	9 (16.1%)	
	Not possible	10 (8.4%)	7 (12.5%)	
Residential status	At home\assisted living	83 (74.8%)	35 (76.1%)	0.965 *
	Nursing home	26 (23.4%)	10 (21.7%)	
	Inpatient Facility	2 (1.8%)	1 (2.2%)	
120-day mortality	Dead	14 (11.2%)	21 (31.3%)	0.001 *
Changes in living situation at 120-day follow-up	Pre-fracture living at home and still living at home	77 (85.6%)	33 (82.5%)	0.903 *
	Pre-fracture living at home changed to nursing home	11 (12.2%)	6 (15.0%)	
	Pre-fracture living at home changed to other inpatient facility	2 (2.2%)	1 (2.5%)	
Readmission to hospital during follow-up	Yes	7 (5.1%)	5 (6.5%)	0.920 *
	No	129 (94.9%)	72 (93.5%)	
Reoperation during follow-up	Yes	6 (4.6%)	5 (6.8%)	0.725 *
	No	124 (95.4%)	68 (93.2%)	
Type of reoperation +	Conversion into total hip arthroplasty	2	0	
	Conversion into hemiarthroplasty	1	0	
	Implant removal	2	0	
	Reposition	0	2	
	Revision of osteosynthesis	1	1	
	Irrigation or debridement	0	2	
	Other	1	1	

* Chi-Square Test; + multiple choices possible.

## 4. Discussion

This study analyzed the surgical management and complication and mortality rate of patients with pathologic hip fractures in contrast to patients with osteoporotic hip fractures. Based on a 2:1 propensity matching, the principal findings revealed that surgical treatment differed significantly between both groups of patients. Patients suffering from pathologic per- and subtrochanteric fractures were more often treated by arthroplasty. In addition, the time to surgery was delayed in patients with pathologic femoral fractures. In terms of survival, an increased mortality rate within the first 120 days of follow-up was seen for pathologic hip fractures according to a multivariate regression analysis. Nevertheless, walking ability and complication rate, as indicated by the rates of reoperation and readmission back to hospital during the 120-day follow-up period, remained unaffected by the fracture type.

Concerning the surgical treatment strategy for pathologic hip fractures, several authors point out the value of an endoprosthetic replacement [22,23,24]. Having conducted a retrospective analysis of 158 patients with pertrochanteric metastatic lesions, Harvey et al. showed that endoprostheses demonstrate a lower mechanical failure rate and a higher rate of implant survivorship without mechanical failure than intramedullary nails [22]. Similar results were published by Steensma et al., who reported the clinical course of 298 patients treated surgically for impending or displaced fractures above the femoral isthmus, excluding the femoral neck. Additionally, in their patients collective, endoprosthetic reconstruction was associated with fewer treatment failures and greater implant durability [23]. Given the results from the above-named literature, it is scarcely surprising that the present registry analysis found a significantly increased rate of arthroplasties performed for per- and subtrochanteric femoral fractures. Nonetheless, with endoprosthetic replacement performed in approximately 9% of cases, the rate of endoprosthetic replacement in ATR-DGU is far below that of Steensma et al., who reported rates between 27 and 41%, depending on the individual fracture site [23].

In contrast to osteoporotic hip fractures, the time to surgery for pathologic hip fractures was significantly delayed in the present registry analysis. While surgical treatment was performed in approximately 80% of patients with osteoporotic hip fractures within the first 24 h, this was the case in only approximately 65% of patients with a pathologic fracture. While delay in time to surgery is known to be directly correlated with mortality in patients with osteoporotic hip fractures, the delay in patients suffering from pathologic femoral fractures was not associated with an increased mortality rate during the acute hospital stay in the present analysis [25]. Therefore, it must be presumed that pathologic hip fractures in geriatric patients are—other than fractures in osteoporosis-related hips—not a typical frailty marker, as is already known from other hip fracture types, e.g., periprosthetic femoral fractures [26].

Even though the mortality rate at the acute hospital stay remained unaffected by fracture type, the results of the present analysis revealed an almost three-fold increased mortality rate for patients suffering from pathologic fractures in the midterm (11.2% vs. 31.3%). Therefore, the results of this present analysis are in line with those of Amen et al., who reported on patients suffering from pathologic hip fractures with a follow-up of 30 days [2]. Based on this elevated mortality rate, Amen et al. concluded that there should be better preoperative patient counseling and shared decision making regarding the decision to undergo surgery at all. According to the results of the present study, it must be presumed that the differences in mortality rate registered among the present follow-up data are mainly driven by the natural course of the disease itself, as the follow-up period is extended up to day 120. Different to Amen et al., we believe that for patients with pathologic hip fractures, surgical fracture fixation is essential to provide adequate pain relief, mobilization and dignity until the end of life. Therefore, we advocate a consequent surgical treatment also in those patients.

In terms of mobilization and complication rates, as indicated by the rates of reoperation and readmission back to the hospital during the 120-day follow-up period, no differences were found between the fracture groups in the present ATR-DGU analysis. Therefore, our results are contradictory to those of Amen et al., who found increased rates of readmission in a 30-day follow-up period for patients with pathologic fractures vs. patients with osteoporotic hip fractures (8.4% vs. 11.9%). Differences in the rate of readmission might be related to the smaller sample size in the present study. Nevertheless, also in our analysis, an at least numerically increased rate of readmission was noticed (4.6% vs. 6.3%). Interestingly, the rates of readmission in the present analysis were much lower than those reported by Amen et al. and Varady et al., although their analyses covered a much shorter follow-up period [2,27]. In this context, it is worth noting that all patients included in this analysis were treated in certified orthogeriatric trauma centers. These centers provide access to orthogeriatric co-management under the best possible conditions that might also cushion the negative effects presumed for patients suffering from cancer-associated as well as osteoporosis-associated hip fractures [28].

### Limitations

Because the present analysis is based on registry data, some limitations must be recognized. While well-designed randomized trials can prove causality, registry analyses, such as the present one, can only describe associations. Our findings must therefore be interpreted with caution. The fact that there is a certain heterogeneity in the patient population included further tempers these findings, as there are different kinds of cancer responsible for the patients subsumed in the group with pathological femoral fractures. Furthermore, due to limitations of the standard documentation sheet thus far, it remains unknown whether the fractures are due to metastases or primary malignant tumors. A possible revision of the standard documentation sheet could allow a more precise statement on this issue in the future.

Despite these above-mentioned limitations, the overall high number of participants included strengthens the results of this registry analysis. Furthermore, with the inclusion of patients from multiple geriatric trauma centers all over Germany, Switzerland and Austria, the present study provides a comprehensive overview of the current treatment strategies and outcomes related to pathologic hip fractures in central Europe.

## 5. Conclusions

The results of the present registry analysis further support current research, as they reveal that outcomes between pathologic and osteoporotic hip fractures are different in terms of surgical treatment strategies, time to surgery and mortality rate in the midterm. The complication rate, as indicated by the rate of readmission to the hospital and the necessity for reoperation, as well as the patients’ walking ability, remained unaffected in the present analysis.

## Figures and Tables

**Table 1:** Univariable analysis of unmatched data on geriatric trauma patients with hip fractures depending on the kind of fracture entity.

Parameter		Non-Pathologic Fracture	Pathologic Fracture	*p*-Value
Number of patients		29,330	211	
Gender	Male	8397 (28.0%)	93 (44.1%)	<0.001 *
	Female	21,081 (72.0%)	118 (55.9%)	
Patient age (year) Mean (sd)		84.4 (6.5)	81.0 (6.7)	<0.001 **
ASA score	1	347 (1.2%)	0 (0.0%)	<0.001*
	2	6489 (22.5%)	26 (12.5%)	
	3	19,780 (68.6%)	147 (70.7%)	
	4 and 5	2201 (7.6%)	35 (16.8%)	
ISAR score	0	2482 (11.3%)	13 (8.2%)	0.161 *
	1	2744 (12.5%)	19 (12.0%)	
	2	4775 (21.8%)	25 (15.8%)	
	3	5244 (23.9%)	40 (25.3%)	
	4	4336 (19.8%)	44 (27.8%)	
	5	1846 (8.4%)	13 (8.2%)	
	6	524 (2.4%)	4 (2.5%)	
Anticoagulatory drugs	Yes	15,387 (54.2%)	93 (45.4%)	0.014 *
	No	12,984 (45.8%)	112 (54.6%)	
Pre-fracture residential status	At home	21,802 (75.6%)	170 (82.1%)	<0.001 *
	Nursing home	6529 (22.7%)	26 (12.6%)	
	Hospital	361 (1.3%)	9 (4.3%)	
	Other	133 (0.5%)	2 (1.0%)	
Fracture type	Hip fracture	13,767 (47.0%)	86 (41.1%)	<0.001 *
	Trochanteric fracture	14,359 (49.0%)	70 (33.5%)	
	Subtrochanteric fracture	1166 (4.0%)	53 (25.4%)	
Time to surgery (h)	<12 h	10,849 (37.3%)	67 (32.2%)	0.002 *
	12–24 h	10,466 (35.9%)	65 (31.2%)	
	24–36 h	3,755 (12.9%)	29 (13.9%)	
	24–48 h	1888 (6.5%)	17 (8.2%)	
	≥48	2157 (7.4%)	30 (14.4%)	
Type of surgical treatment +	Total hip arthroplasty	2389	24	
	Hemiarthroplasty	10,136	65	
	Trochanteric nail	14,742	102	
	Dynamic hip screw	929	10	
	Cannulates screw	381	2	
	Other	913	11	
Pre-fracture walking ability	Independent without walking aids	9610 (35.1%)	57 (29.1%)	0.158 *
	Ability to walk outside with a walking stick or crutch	3296 (12.1%)	34 (17.3%)	
	Ability to walk outside with two crutches or a walker	8882 (32.5%)	64 (32.7%)	
	Certain walking ability in the apartment, but outside only with an assistant	4694 (17.2%)	35 (17.9%)	
	No functional walking ability	869 (3.2%)	6 (3.1%)	
Death during stay in the acute hospital	Yes	1622 (5.5%)	22 (10.4%)	0.065 *
	No	27,649 (94.5%)	189 (89.6%)	
Ability to walk at the seventh postoperative day	Unknown	842 (2.9%)	4 (1.9%)	0.602 *
	Without aid	182 (0.6%)	3 (1.4%)	
	With walking stick or crutch	3106 (10.7%)	22 (10.6%)	
	With a rollator	8561 (29.5%)	67 (32.2%)	
	With a walking frame (no wheels)	4043 (13.9%)	29 (13.9%)	
	With a walker	6282 (21.7%)	38 (18.3%)	
	Not possible	5994 (20.7%)	45 (21.6%)	
Reoperation during initial acute hospital stay	Yes	964 (3.3%)	9 (4.3%)	0.550 *
	No	28,340 (96.7%)	202 (95.7%)	
Discharge from hospital	At home	6774 (24.8%)	57 (31.0%)	0.002 *
	Nursing home	7367 (27.0%)	38 (20.7%)	
	Inpatient stay	13,151 (48.2%)	89 (48.3%)	

* Chi-Square Test; ** Mann–Whitney; + multiple choices possible.

**Table 2:** Univariable analysis of a 2:1 optimal propensity score matching analysis of data on geriatric trauma patients with hip fractures depending on the kind of fracture entity.

Parameter		Non-Pathologic Fracture	Pathologic Fracture	*p*-Value
Number of patients		382	191	
Gender	Male	180 (47.1%)	83 (43.5%)	0.459 *
	Female	202 (52.9%)	108 (56.5%)	
Patient age (year) Mean (sd)		81.1 (6.6)	81.1 (6.7)	0.968 **
ASA score	2	41 (10.7%)	24 (12.6%)	0.703 *
	3	273 (71.5%)	137 (71.7%)	
	4	68 (17.8%)	30 (15.7%)	
ISAR score	0	24 (8.7%)	13 (9.0%)	0.420 *
	1	27 (9.8%)	19 (13.2%)	
	2	67 (24.3%)	22 (15.3%)	
	3	71 (25.7%)	36 (25.0%)	
	4	60 (21.7%)	39 (27.1%)	
	5	23 (8.3%)	12 (8.3%)	
	6	4 (1.4%)	3 (2.1%)	
Anticoagulatory drugs	Yes	225 (60.3%)	85(45.5%)	0.001 *
	No	148 (39.7%)	102 (54.5%)	
Pre-fracture residential status	At home	298 (79.3%)	153 (81.0%)	0.105 *
	Nursing home	68 (18.1%)	26 (13.8%)	
	Hospital	5 (1.3%)	8 (4.2%)	
	Other	5 (1.3%)	2 (1.1%)	
Fracture type	Hip fracture	163 (42.7%)	79 (41.4%)	0.814 *
	Trochanteric fracture	138 (36.1%)	67 (35.1%)	
	Subtrochanteric fracture	81 (21.2%)	45 (23.5%)	
Time to surgery (h)	<12 h	151 (39.7%)	62 (33.0%)	0.005 *
	12–24 h	152 (40.0%)	60 (31.9%)	
	24–36 h	31 (8.2%)	27 (14.4%)	
	36–48 h	21 (5.5%)	16 (8.5%)	
	≥48	25 (6.6%)	23 (12.2%)	
Type of surgical treatment +	Total hip arthroplasty	45	20	
	Hemiarthroplasty	109	60	
	Trochanteric nail	212	94	
	Dynamic hip screw	9	9	
	Cannulates screw	4	1	
	Other	10	9	
Type of surgical treatment for per- and subtrochanteric fractures	Total hip arthroplasty or hemiarthroplasty	3 (1.3%)	10 (8.8%)	0.002 *
	Osteosynthesis	221 (98.7%)	104 (91.2%)	
Pre-fracture walking ability	Independent without walking aids	106 (27.7%)	53 (27.7%)	0.892 *
	Ability to walk outside with a walking stick or crutch	62 (16.2%)	34 (17.8%)	
	Ability to walk outside with two crutches or a walker	127 (33.2%)	63 (33.0%)	
	Certain walking ability in the apartment, but outside only with an assistant	79 (20.7%)	35 (18.3%)	
	No functional walking ability	8 (2.1%)	6 (3.1%)	
Death during stay in the acute hospital	Yes	25 (6.6%)	19 (9.9%)	0.756 *
	No	356 (93.4%)	172 (90.1%)	
Ability to walk at the seventh postoperative day	Unknown	6 (1.6%)	4 (2.1%)	0.198 *
	Without aid	4 (1.1%)	2 (1.1%)	
	With walking stick or crutch	36 (9.5%)	22 (11.6%)	
	With a rollator	120 (31.7%)	61 (32.3%)	
	With a walking frame (no wheels)	37 (9.8%)	29 (15.3%)	
	With a walker	103 (27.2%)	34 (18.0%)	
	Not possible	72 (19.0%)	37 (19.6%)	
Reoperation during initial acute hospital stay	Yes	10 (2.6%)	8 (4.2%)	0.446 *
	No	372 (97.4%)	183 (95.8%)	
Discharge from hospital	At home	92 (25.9%)	54 (32.3%)	0.202 *
	Nursing home	86 (24.2%)	36 (21.6%)	
	Inpatient stay	177 (49.9%)	77 (46.1%)	

* Chi-Square Test; ** Mann–Whitney; + multiple choices possible.

## Data Availability

Not applicable.
